# Topically Delivered Treg‐Attracting Microparticle‐Hydrogel Composites Recruit Regulatory T Cells and Limit Cardiac Injury After Severe Ischemia

**DOI:** 10.1002/adhm.71861

**Published:** 2026-10-18

**Authors:** Matthew A. Borrelli, Alexis T. Reese, Jordan J. P. Warunek, Betsy Ann Varghese, Canxing Lin, Jeffery J. Baust, Brenda McMahon, Steven R. Little, Hēth R. Turnquist

**Affiliations:** ^1^ Department of Chemical and Petroleum Engineering University of Pittsburgh Pittsburgh PA USA; ^2^ Thomas E. Starzl Transplantation Institute University of Pittsburgh School of Medicine Pittsburgh PA USA; ^3^ Department of Surgery University of Pittsburgh School of Medicine Pittsburgh PA USA; ^4^ Department of Immunology University of Pittsburgh School of Medicine Pittsburgh PA USA; ^5^ Division of Pulmonary Allergy and Critical Care Medicine Department of Medicine University of Pittsburgh School of Medicine Pittsburgh PA USA; ^6^ Ultrasonography Department Mount Aloysius College Cresson PA USA; ^7^ Department of Bioengineering University of Pittsburgh Pittsburgh PA USA; ^8^ Department of Pharmaceutical Sciences University of Pittsburgh Pittsburgh PA USA; ^9^ Department of Ophthalmology University of Pittsburgh Pittsburgh PA USA; ^10^ Clinical and Translational Science Institute University of Pittsburgh Pittsburgh PA USA; ^11^ McGowan Institute For Regenerative Medicine University of Pittsburgh PA USA

**Keywords:** hydrogel, Ischemic Heart Injury, microparticles, myocardial infarction, Treg

## Abstract

Ischemic heart disease is a highly prevalent and debilitating condition throughout the world. Following an ischemic injury, the immune response can become overly active and spread damage to nearby healthy tissue. Because the heart lacks regenerative capacity, preventing excessive damage is critical. Existing immunosuppressive therapeutics provide indiscriminate suppression of inflammatory and repair functions of infiltrating immune cells. Regulatory T cells (Treg) are the body's highly sophisticated system for regulating immunologic activity. These cells can provide powerful immune suppression while amplifying repair pathways through direct stimulation of local cells. Our research group has previously developed a polymeric delivery system comprised of poly(lactic‐co‐glycolic acid) (PLGA) degradable microparticles (MP) loaded with the Treg‐attracting protein, CCL22. In our previous work, we demonstrated CCL22MP attracts Treg to the injection site and can suppress disadvantageous inflammation. Here we combine our Treg‐attracting MP technology with **H**ydrogels to **E**nhance or **A**ttract **R**egulatory **T** cells (**HEART**). Topically applied HEART significantly reduced infarct size and improved ejection fraction after permanent‐ligation MI, and in a cardiac transplant ischemia model reduced graft fibrosis while significantly increasing intragraft Foxp3^+^ Treg at 30 days. Efficacy tracked with injury severity, identifying severe ischemic injury as a setting where this therapeutic strategy may be most beneficial.

## Graphical abstract

CCL22‐releasing PLGA microparticles (MP) suspended in topically applied hydrogels (HEART) establish a sustained chemokine gradient at the injured myocardium. HEART recruits CCR4^+^ regulatory T cells, reduces infarct size and graft fibrosis, and improves cardiac function. Benefit tracks with injury severity, being greatest after severe ischemic events.

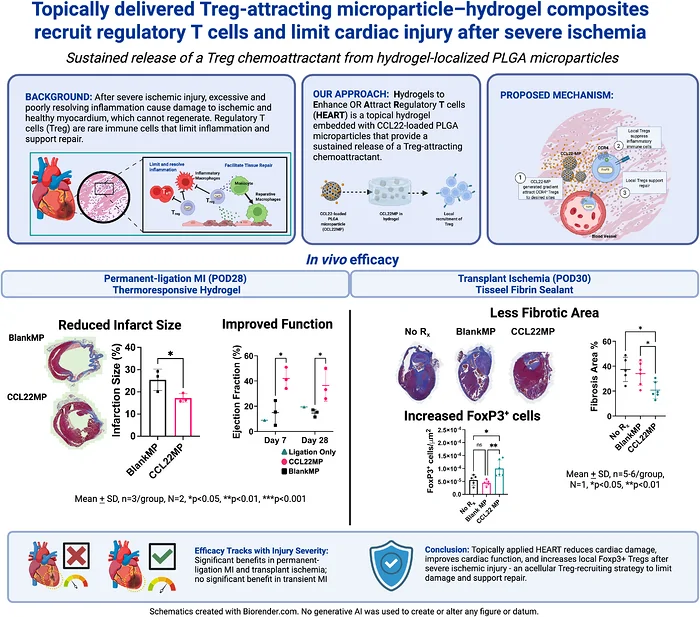

## 1 Introduction

Cardiovascular disease can be caused by a myriad of disorders, but cardiac ischemia, called myocardial infarction (MI), is a key triggering event. When MI occurs, early tissue injury and later pathologic tissue remodeling result in rapid progression of cardiovascular disease [1]. Each year, ischemic heart disease can be linked to approximately 9 million deaths [2]. For those that survive their first MI, approximately 18.8% of patients are readmitted within 90 days [3]. Moreover, MI survivors often develop ischemic heart disease and progress to clinical heart failure within a few years [4]. Consequently, MI and related hospitalization or treatment costs place a considerable burden on the health care system [2]. Worldwide, 250 million individuals live with ischemic heart disease [2], thus better solutions are sorely needed to address this disease.

Ischemic injury instigates significant damage to the heart that existing therapeutic strategies do not effectively treat. Ischemic injury is caused by blocked or reduced coronary blood flow, which creates localized hypoxia and induces necrosis of cardiomyocytes (CMs). The area of this hypoxic injury is commonly called an infarct. CM necrosis releases damage‐associated molecular patterns (DAMPs) that cause inflammation and activate tissue‐resident cell types involved in repair. Unlike other tissues in the body, the heart lacks the ability to regenerate functional CMs, thus any damage is permanent [5]. Longer periods between ischemia and reestablishment of blood flow (reperfusion) will create larger infarcts [6, 7], which significantly elevates the chances of developing into ischemic heart disease. Accordingly, the clinical standard for treating MI patients includes rapid reperfusion via percutaneous coronary intervention (PCI) or thrombolytics [7], which are frequently supported by vasodilators [8, 9]. While beneficial in the short term, reperfusion also causes damage to the heart, referred to as reperfusion injury (RI). RI may account for up to half of the final infarct size [10]. Importantly, there are no FDA‐approved therapies that can prevent or reduce the severity of RI. Additionally, research efforts to identify strategies to address RI have struggled to translate into optimal clinical benefits, as reviewed by Alsadder et al. [1]. A key challenge of RI is that multiple cellular processes are dysregulated in response to ischemic injury, requiring candidate therapies to address several pathways at once [11, 12].

The immune system, which responds to injury and orchestrates repair, often becomes dysregulated following cardiac ischemia through poorly understood mechanisms [10]. DAMPs, also referred to as “alarmins,” are endogenous molecules—including high‐mobility group box 1 (HMGB1), extracellular ATP, heat‐shock proteins, interleukin‐1α (IL‐1α), and IL‐33 – that are typically sequestered in healthy cells and released upon cellular stress or necrosis to signal tissue damage [13, 14, 15]. DAMPs released by necrotic CMs attract and activate circulating immune cells. Indiscriminate activation of infiltrating immune cells by DAMPs causes uncontrolled inflammatory signaling and effector actions (e.g. NETosis, oxidative species, granzymes) that damage neighboring healthy cardiac tissue, which is called infarct spreading [10, 16]. This overactivation and injury continues for several days until shifting toward a pro‐wound healing response. The magnitude of the initial immune response and the way that it ultimately resolves appears to significantly shape the repair and remodeling of the tissue [17]. Importantly, inflammation often does not adequately resolve following significant cardiac injuries. Complete understanding for ineffective resolution remains elusive, but on‐going stimulation of immune effector cells, ineffective apoptosis of effector cells, or persistent cellular debris from apoptotic and necrotic bodies have been implicated [18, 19]. Indeed, persistent environmental cues in the infarcted tissue appear to perpetuate inflammatory phenotypes of local immune cells [16, 20].

Consequently, several studies have aimed toward understanding the immune response following MI [16, 21, 22, 23, 24] and subsequent heart failure [24, 25]. Mechanistic studies in transgenic genetic knockout models have implicated Foxp3^+^CD4^+^ Regulatory T cells (Treg) [26, 27] and their secreted factors [28, 29, 30], as critical mediators of inflammation and regulators of repair in the infarcted heart. Additionally, there is evidence that systemic administration of a bolus dose of a CXCR4 antagonist can also reduce cardiac injury after MI [31]. CXCL12 chemokine binding CXCR4 has been shown to mobilize endothelial progenitor cells [32], but it was also reported that administration of the CXCR4 antagonist enhanced leukocyte migration into the circulation, including neutrophils, monocytes, and Treg. Because this antagonist was administered as systemic bolus dose, the effects were transient. Additionally, this approach lacks specificity due to the broad expression of CXCR4 in the hematopoietic system [33]. Therefore, sustained chemoattraction of Treg by using a more selective chemokine/receptor may have greater potential to provide increased benefit.

In our prior work, we have developed a Treg‐recruiting microparticle (MP) formulation that provides a sustained gradient of the Treg‐targeting chemokine, CCL22 (CCL22MP) [34]. CCL22 signals through the CCR4 receptor, which is highly enriched on Treg, but also expressed at varying levels on Th2, as well as Th17, NK, monocyte/macrophages and dendritic cell populations [35, 36]. Prior studies have demonstrated that CCL22MP treatment in disease models characterized by an unwanted or disproportionate immune response that does not effectively resolve (e.g., Transplant [37], Dry‐eye disease [38], periodontitis [39], skeletal muscle injury [40]) increases local Treg populations and diminishes signs of disease. In each of these models, direct injection of CCL22MP into the dermal tissue could provide effective localization and retention of the MP to the site of injury, but adapting this therapy for cardiac delivery requires innovation.

In this work, we endeavored to translate our CCL22MP platform to be suitable for cardiac delivery. We accomplished this by suspending CCL22MP in a variety of hydrogels, thereby combining sustained and controllable release rates from polymeric MP with tissue‐conforming, injectable, and bulking properties of hydrogels [41, 42, 43]. Specifically, we evaluate three distinct hydrogel platforms across the iterative stages of our study: first, a nondegrading and thermally responsive hydrogel; second, an injectable hydrogel made from functionalized hyaluronic acid; and finally, four tissue adhesive hydrogels—a photocrosslinked HEMA‐hyaluronic acid gel and the FDA‐approved sealants Coseal, Artiss, and Tisseel—screened to identify the one best suited for in vivo application. We next evaluate how these combinatory **H**ydrogels to **E**nhance or **A**ttract **R**egulatory **T** cells (**HEART**) may affect the extent of injury to the heart following ischemia. Because the heart does not regenerate, we hypothesize that the key potential for benefit to the injured heart is improved control of infarct spreading and that greater injury will result in greater benefit. Accordingly, we induced ischemic injury using three different models that would result in distinct levels of damage to the heart. Specifically, we employed a transient ischemia RI model of MI (limited local damage), a chronic ischemia model of MI (significant local damage), and a transplant ischemia model (significant global damage). We find that HEART provides a significant reduction to cardiac injury in chronic ischemia and transplant ischemia, but not short‐term ischemia‐RI. Based on these results, HEART therapy may provide the greatest benefit to patients who sustained a severe cardiac injury or did not receive care in a timely manner. Continued development of HEART could ultimately contribute to improved cardiac care and reduce the progression to post‐ischemic heart failure.

## 2 Methods

### 2.1 Materials

RG502H Poly(D,L‐lactic‐co‐glycolic acid) (PLGA) polymer, with 50:50 lactide:glycolide composition was obtained from Evonik (Essen, Germany). Poly(ethylene glycol) (PEG) terminated PLGA (PEG‐PLGA), and Poly(vinyl alcohol) (PVA, 98 mol% hydrolyzed, *M_w_* = 25000 g mol^−1^) were purchased from PolySciences (Warrington, PA). Carrier‐free recombinant murine CCL22 (mCCL22) was obtained from R&D systems (Minneapolis, MN). Bovine Serum Albumin (BSA) was obtained from Fisher Chemical (Waltham, MA). Dichloromethane (DCM), Acetonitrile (ACN), Sodium Dodecyl Sulfate (SDS), was obtained from Fisher Chemical (Waltham, MA). Phosphate buffered saline (PBS) was obtained from Fisher Scientific (Waltham, MA).

### 2.2 Microparticle (MP) Formulation

Double emulsion microparticles (MPs) encapsulating CCL22 or blanks were formulated as previously described [34, 44]. Briefly, the inner, aqueous phase (200 µL vol) contains CCL22 protein (5 µg) (CCL22MP) or water (BlankMP), 0.375 mg of protein‐stabilizing bovine serum albumin (BSA), and 15 mOsm of NaCl to produce surface pores. The outer, organic phase contains PLGA polymer (200 mg, 55 wt.% PEG‐PLGA & balance RG502H) solvated in dichloromethane (DCM) (4 mL). The inner phase is emulsified (water‐in‐oil, w/o) with the outer phase via probe sonication (Active Motif EpiShear probe sonicator, 110 V) at 55% amplitude for 10 s and then transferred to a 2% PVA solution (60 mL) and homogenized (L4RT‐A; Silverson, East Longmeadow, MA) at 3000 rpm for 1 min, forming the second emulsion (w/o/w). The emulsified solution was transferred to a 1% (w/v) PVA solution (80 mL) and stirred at 600 RPM for 3 h at 4°C. Freshly formed MPs were centrifuged (Eppendorf 5810R 15‐amp version, 200 xg for 5 min at 4°C) and washed four times (4x) with 4°C MilliQ water (Milli‐Q IQ 7000, Millipore Sigma) (35 mL). The MPs were then re‐suspended in MilliQ water (5 mL), flash‐frozen with liquid nitrogen (5 min), and lyophilized (Benchtop Pro, Virtis SP Scientific) (< 100 mTorr, 48–72 h). The aqueous phase of fluorescently labeled MP created for in vivo particle tracking contained 125 µg of AlexaFluor680‐labeled dextran (M_w_ = 10 kDa) solvated in 200 µL of DI water. All other fabrication steps proceeded the same.

### 2.3 Hydrogel Formulation

Thermogels were prepared via aqueous free radical polymerization. Briefly, 100 µL of PEG (MW 200 da), 100 mg of NIPAAm, and 0.2 mg ammonium persulfate were solvated in 2 mL of DI water. This mixture was vortexed until the solution was homogeneously mixed. Tetramethylethylenediamine (5 µL) was added to initiate the polymerization reaction, which continued overnight at 4°C. The resulting polymer was washed 5 times using DI water at 45°C. Shear‐thinning hydrogels were formed from functionalized hyaluronic acid, as previously described and characterized [45]. Briefly, hyaluronic acid was functionalized with adamantane (HA‐AD) and cyclodextrin (HA‐CD) (20% and 18% functionalization, respectively). HA‐AD and HA‐CD were resuspended (4% wt./vol total HA) in PBS with MPs at the specified concentration and mixed in a 1 mL syringe. Guest‐host interactions between the adamantane and cyclodextrin form spontaneously to yield the hydrogel, and these interactions break under shear stress resulting in an injectable hydrogel. *UV‐curing hydrogel* was supplied by Dr. Jason Burdick at the University of Colorado, Boulder. This gel was created by functionalizing hyaluronic acid with hydroxyethyl methacrylate (HEMA‐HA, 20% modification), in which the HEMA groups polymerize in exposure to ultraviolet light [46]. This hydrogel has previously been characterized and used for cardiac localized delivery [47, 48]. HEMA‐HA (6 wt.% in water) solution was photopolymerized with UV exposure from a halogen curing light (Aphrodite, 1500 MW) for 2 min to form the hydrogel. FDA‐approved hydrogels were obtained from Baxter (Deerfield, IL), including COSEAL, ARTISS, and TISSEEL with slight modifications to gelation initiator concentrations to achieve gelation 60 s after mixing. COSEAL gel was formed from PEG‐NHS powder solvated in HCl (pH2) combined with 0.2 M Na_2_PO_4_ (pH 7) in equal volume for a total volume of 100 µL. TISSEEL and ARTISS gels are formed from fibrinogen/thrombin interaction by mixing equal volumes of a fibrinogen solution and thrombin solution. For ARTISS and TISSEEL, the fibrinogen solution consisted of Human fibrinogen (86.5 mg/mL) and fibrinolysis inhibitor, aprotinin 3,000 KIU/mL. The thrombin solution consisted of PBS supplemented with CaCl_2_ (40 µmol/mL) and 4 or 25 U/mL of Thrombin, for ARTISS and TISSEEL, respectively. Unless otherwise stated, MP were suspended at a concentration of 10 mg/mL relative to cross‐linked hydrogel volume.

### 2.4 Microparticle Release Assays

To determine MP in vitro release behavior, 10 mg of MPs were solvated in 1 mL of 1% (w/v) bovine serum albumin (BSA) in PBS and incubated on a roto‐shaker (Thermo Scientific Tube Revolver Rotator, 1.5–2 mL Eppendorf tube paddles, speed 10) at 37°C. At specified time intervals, MP suspensions were centrifuged (580 g for 5 mins at room temperature) (Eppendorf 5417R), 800 µL of the supernatant was removed and replaced with 800 µL of fresh release media, and the MPs were resuspended and placed back on the roto‐shaker. Supernatant concentrations of CCL22 were determined via enzyme‐linked immunosorbent assay (ELISA; R&D Systems), and subsequently the ELISA results were interpreted through quantification of optical density (SpectraMax M5; Molecular Devices; Sunnyvale, CA). Release profiles generated from measured concentrations of CCL22 had any background signal from BlankMP subtracted and then normalized to the total amount loaded within MPs (quantified by solvating MPs and extracting the protein liberated) and MP mass.

### 2.5 Langendorff Apparatus

Male C57BL/6J mice aged 8–14 weeks were purchased from Jackson labs (Bar Harbor, ME). Hearts were excised from healthy WT mice, flushed with PBS + heparin, attached to a Langendorff apparatus, and perfused on working heart mode to evaluate the effect of hydrogel injection on cardiac function. Cardiac function was measured for 5 min prior to injection of hydrogel and for 5 min following hydrogel injection.

### 2.6 Microparticle Retention Study

Wistar rats (aged 8–12 weeks) were purchased from Jackson Labs (Bar Harbor, ME). The rats were anesthetized with 2% isoflurane in oxygen at a flow rate of ∼2 L/min and intubated. Thoracotomy was performed to expose the heart and injectable hyaluronic acid gels, containing AF680 Dextran MP (20 mg/mL) or controls (BlankMP, no MP), were injected (100 uL) into the myocardium of healthy Wistar rats. The chest was closed and the animals were allowed to recover on a heated pad (37°C) before being transferred to a clean cage. After 14 days, the animals were euthanized via inhalation of CO_2_ and the hearts were excised. Fluorescence in the hearts was quantified using an IVIS Lumina XR operated by Living Image software version 4.3.1. Fluorescent intensity analysis was then conducted in ImageJ.

### 2.7 Transplant Ischemia Model

Syngeneic heart transplants were conducted using age‐matched 8‐12‐week‐old C57BL/6J male mice donors and recipients. Donor hearts were subjected to 4‐hour ischemia prior to transplantation. Ischemic donor hearts were transplanted into recipients through end‐to‐side anastomosis of the donor ascending aorta and pulmonary artery to recipient abdominal aorta and inferior vena cava, as described previously [49]. Upon reperfusion of the transplanted heart, mice receiving BlankMP or CCL22MP were administered 100 µL of TISSEEL hydrogel with 10 mg/mL of suspended MP, topically to the heart. The hydrogel volume was strategically targeted to the visible cardiac surface area to cover the region of ischemic injury. The abdomen was closed, 2.5 µL/g Ethiqa was subcutaneously injected, and mice were placed on a heated pad (37°C) to recover before being transferred to a clean cage. Mice were monitored every 1–2 days post‐transplantation and euthanized if they exhibited any of the following humane endpoints: >20% weight loss, inability to ambulate, unresponsiveness to external stimuli, a moribund condition, or incision site infection. After 30 days, the mice were euthanized via inhalation of CO_2_ gas, and the hearts were harvested for histologic quantification of fibrotic tissue deposition and FoxP3^+^ cells. To evaluate the early immune response, an additional cohort of transplant recipient mice was assessed on post‐operative day 5 (POD 5). Prior to euthanasia, these mice received a 3 µg intravenous injection of a fluorochrome‐conjugated CD45 antibody to label circulating leukocytes. After 3 min, the mice were euthanized via CO_2_ inhalation, and donor hearts were harvested for flow cytometric quantification of immune cell populations.

### 2.8 Myocardial Infarction Models

For myocardial infarction procedures, B6 WT mice (age 8–12 weeks) were purchased from Jackson Labs. MI was induced via ligation of the left aortic descending (LAD) artery permanently (Thermogel Experiments) or transiently (30 mins) (Injectable Hydrogel Experiments). This method of myocardial infarction simulates clinical presentation of the disease and produces a reproducible infarction in the left ventricle (LV). More information can be found in JOVE [50]. Immediately following LAD arterial ligation, the respective hydrogel with 10 mg/mL MP suspended were administered. The thermogel was administered topically (100 µL), proximal to the infarcted myocardium, while the injectable hydrogel was injected into the infarct border zone (20 µL). The chest was then closed, and mice were placed on a heating pad (37°C) to recover before being transferred to a clean cage. Mice were monitored until study conclusion on Postoperative Day 28, when the animals were euthanized via CO_2_ gas inhalation and the hearts were harvested for histologic assessment.

### 2.9 Echocardiography

On day 7 and 28 after MI induction, transthoracic echocardiography was performed using a VisualSonics Vevo 3100, a high‐frequency ultrasound machine with the MS 400 transducer (VisualSonics Inc). The mice were anesthetized with isoflurane at 3% induction. During the echocardiogram, isoflurane was reduced to 1.5% to maintain a heart rate greater than 400 bpm. A short axis image of the LV (SAX) was obtained, and an M‐mode image was taken at mid ventricular, with evidence of the papillary muscles. A parasternal long axis of the left ventricle (PSLAX) was also acquired. Analysis was then completed with VevoLab Software provided by VisualSonics Inc. An average of at least 3 cardiac cycles was analyzed in all measurements. The SAX M‐Mode image was used to obtain heart rate, ventricular wall thickness, ventricular cavity size, ejection fraction, fractional shortening and cardiac output. To acquire the volume of the left ventricular cavity in PSLAX, the left cavity area was measured in systole and diastole.

### 2.10 Tissue Collection and Histologic Assessment of Fibrosis

Upon transplant ischemia study and MI studies’ conclusion, experimental C57BL/6 wild‐type mice were euthanized, and the hearts were perfused with 1% heparin in PBS and fixed for histologic processing. For the MI studies, transverse tissue sections (10 µm thickness) were prepared from the apex to the base of the LV; for the transplant ischemia study, the heart was bisected longitudinally and three, 10 µm sections were taken. Sections were stained using Masson's Trichrome Staining, in which the blue‐stained portions of the tissue represent areas of fibrosis and collagen deposition, and the red sections represent healthy cardiac tissue. Ratios of fibrotic, infarcted tissue to the healthy cardiac tissue were quantified using ImageJ. Additional sections from the transplant ischemia study were immunostained for FoxP3, subjected to whole slide imaging, and analyzed in QuPath using a custom‐trained cell classifier to quantify FoxP3^+^ cells.

### 2.11 Flow Cytometry

For the POD5 transplant ischemia study, transplanted hearts were minced and homogenized using a gentleMACS Dissociator in 10 mL of RPMI, supplemented with 2% FBS, 250 µL collagenase IV (50 mg/mL), and 10 µL DNAse I (20 mg/mL). The resulting digests were passed through a 70 µm cell strainer and purified using a 37.5% Percoll density gradient. Isolated cells from hearts were stained with fluorochrome‐conjugated antibodies, acquired on an Aurora (Cytek Biosciences), and analyzed using FlowJo software.

### 2.12 Flow Cytometry Antibodies

Zombie UV Fixable Viability (1:500, BioLegend), BV421 anti‐mouse CD45.1 (1:200, Invitrogen, Clone: A20), PerCP‐Cy5.5 anti‐mouse CD45.2 (1:150, BD Biosciences, Clone 104), APC‐eFluor780 anti‐mouse CD11b (1:200, Invitrogen, Clone: M1/70), BV510 anti‐mouse CD11c (1:200, BioLegend, Clone: N418), Pacific Blue anti‐mouse F4/80 (1:100, BioLegend, Clone: BM8), BUV805 anti‐mouse Ly6G (1:200, BD Biosciences, Clone: 1A8), AF700 anti‐mouse CD3 (1:150, BD Biosciences, Clone: 17A2), BUV563 anti‐mouse CD4 (1:150, BD Biosciences, Clone: GK1.5), PE‐Cy5 anti‐mouse B220 (1:200, BD Biosciences, Clone: RA3‐6B2), PE anti‐mouse Ly‐6C (1:200, BD Biosciences, Clone: AL‐21), BV650 anti‐mouse CD86 (1:200, BioLegend, Clone: GL‐1), BUV395 anti‐mouse MHCII (1:200, BD Biosciences, Clone: 2G9), BV785 anti‐mouse CD25 (1:100, BioLegend, Clone: PC61), BUV737 anti‐mouse ST2 (1:100, BD Biosciences, Clone: U29‐93), Pacific Orange anti‐mouse CD45 (3 µg I.V., Invitrogen, Clone: 30‐F11), FITC anti‐mouse FoxP3 (1:100), PE‐Cy7 anti‐mouse iNOS (1:300, Invitrogen, Clone: CXNFT), BV605 CD206 (1:200), PE‐Dazzle 594 anti‐mouse CD301b (1:200, BioLegend, Clone: URA‐1), and PerCP‐eFluor710 anti‐human/mouse Arginase 1 (1:100, Invitrogen, Clone: A1exF5).

### 2.13 Statistics

CCL22MP release experiments were performed in triplicate, and data represent means with standard deviation indicated with error bars. For single comparisons, the data were checked for normality and a double‐sided student's T test was employed. For multiple comparisons, One‐Way ANOVA was performed followed by Šídák's multiple comparisons test. Differences in means were considered to be significant if p ≤ 0.05. Animal numbers were not derived from a formal a priori power calculation; instead, group sizes were set from the effect sizes and variances reported in comparable cardiac transplant and myocardial injury studies from our two laboratories and from the published literature [51, 52, 53] using the smallest cohort expected to resolve the primary endpoint. Most mechanistic and characterization studies used n = 3–4 animals per group and outcome studies typically used n = 5–9 per group; the exact number for each experiment is given in the corresponding figure legend.

### 2.14 Sex as a Biological Variable

Only male mice were used in this study to maintain statistical power across multiple experimental arms given the number of timepoints, injury models, and treatments assessed. For the syngeneic transplant ischemia model, sex‐matched (male) donor/recipient pairs also avoided introducing an unintended H‐Y minor histocompatibility antigen mismatch into this otherwise syngeneic, nonalloimmune model.

### 2.15 Study Approvals

Both surgical models are survival procedures performed under isoflurane anesthesia with extended‐release buprenorphine analgesia (Ethiqa XR, 2.5 µL/g s.c.) and are classified under USDA Pain and Distress Category D. Refinement measures such peri‐operative warming, monitoring every 1–2 days, and predefined humane endpoints are described above. All procedures were completed at the University of Pittsburgh and were approved by the IACUC of the University of Pittsburgh and complied with the NIH's Guide for the Care and Use of Laboratory Animals (National Academies Press, 2011). The MI model protocol (#24085489) was approved on 8/21/2024, and the transplant ischemia protocol (#25056502) was approved on 5/13/2025.

## 3 Results

### 3.1 Pilot Study Localizing CCL22MP To the Ischemic Heart with A Thermally Responsive Hydrogel Shows Efficacy

To determine if CCL22MP treatment would provide a benefit following MI, a preliminary study in a permanent LAD ligature model of MI was conducted. In this pilot study, CCL22MP (see Figure 1B for the release profile) or BlankMP were suspended in a thermally responsive hydrogel, and the gel and MP solution was then applied, topically, to the infarcted heart. Figure 1A shows the study scheme and delineates the approximate timeline for the three phases of the cardiac injury response. A significant reduction in mean infarction size of ∼10% was observed between CCL22MP and BlankMP groups (Figure 1C,D). Significant improvements in ejection fraction (POD 7 and 28), systolic volume (POD 7 and 28), and diastolic volume (POD 28) were observed in the CCL22MP group compared to the Blank MP group (Figure 1E). These data suggest that CCL22MP treatment following MI can improve heart function by reducing tissue damage.

**FIGURE 1:**
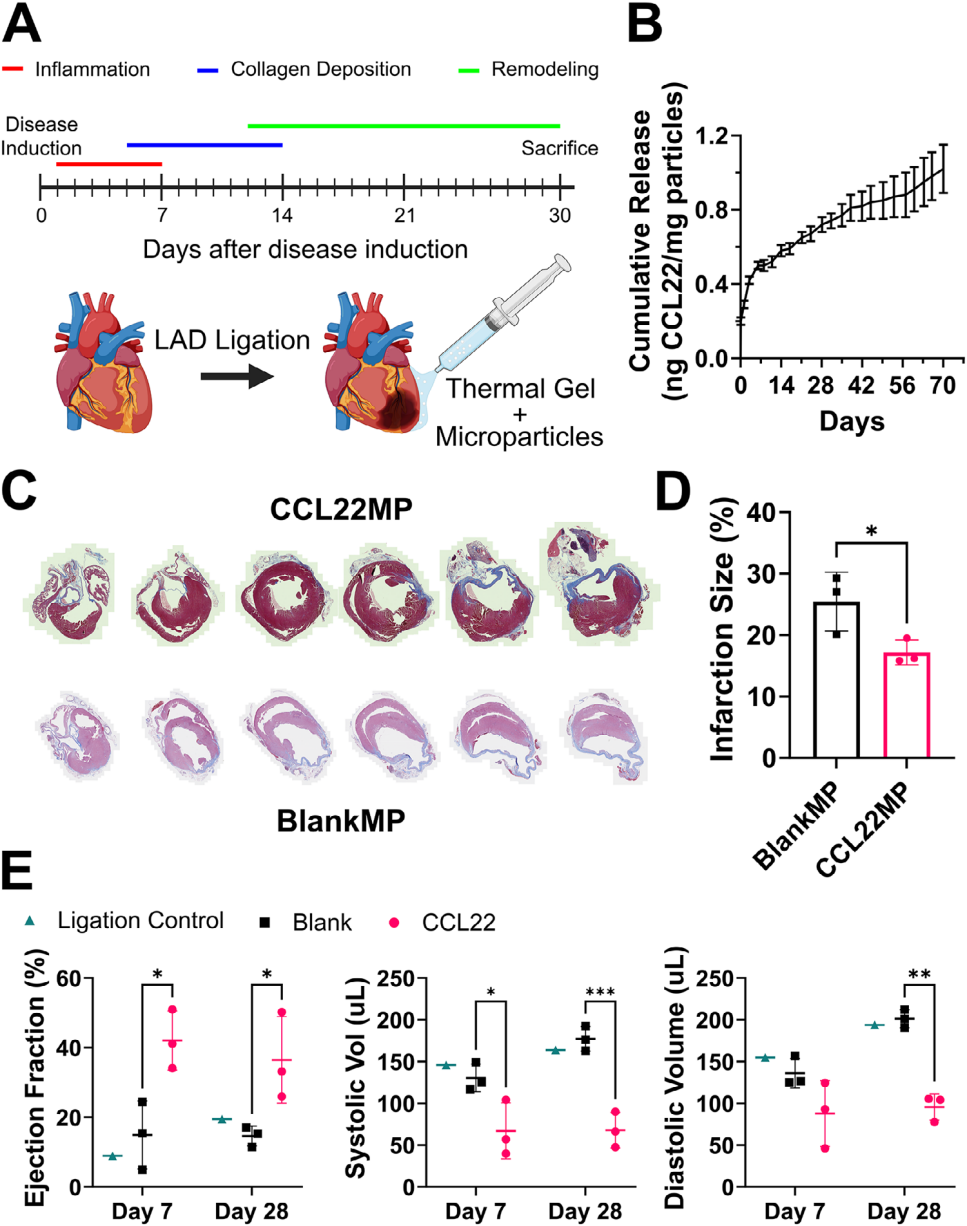
Topical delivery of a nondegradable thermally responsive hydrogel loaded with CCL22MP reduces myocardial infarction injury. To understand the potential benefit from CCL22MP treatment in cardiac injury, we performed a proof‐of‐concept pilot study in a permanent ligature model of MI. (A) Study scheme. Created with BioRender.com. (B) Cumulative release of CCL22 from CCL22MP shows sustained release for up to 70 days following an initial burst in the first week of release. (C) Representative serial trichrome‐stained histology cross‐sections (n = 6 sections/heart, from apex to base) for one BlankMP and one CCL22MP‐treated heart harvested 30 days after MI (n = 3 mice/group). (D) Quantification of the collagen (Infarct) as a percentage of the healthy myocardial tissue, averaged across all 6 sections per heart (n = 3 mice/group). (E) Cardiac functional measurements were quantified via echocardiography, including ejection fraction, systolic volume, and diastolic volume at day 7 and 28 (n = 3/treatment group; n = 1 ligation control shown as reference). Data are mean ± SD. *p < 0.05, **p < 0.01, ***p < 0.001.

### 3.2 CCL22MP Formulated Into a Clinically Translatable, Injectable Hydrogel

Following positive results using a nondegradable and thermally responsive hydrogel, we sought to formulate a clinically translatable **i**njectable platform for our hydrogel to enhance or attract regulatory T cells (**iHEART**). We observed depressed, but more linearized release of CCL22 following suspension of CCL22MP in a hyaluronic acid‐based hydrogel (Figure 2A). Following lyophilization, iHEART was found to fully re‐hydrate after 3 h (Figure 2B).

**FIGURE 2:**
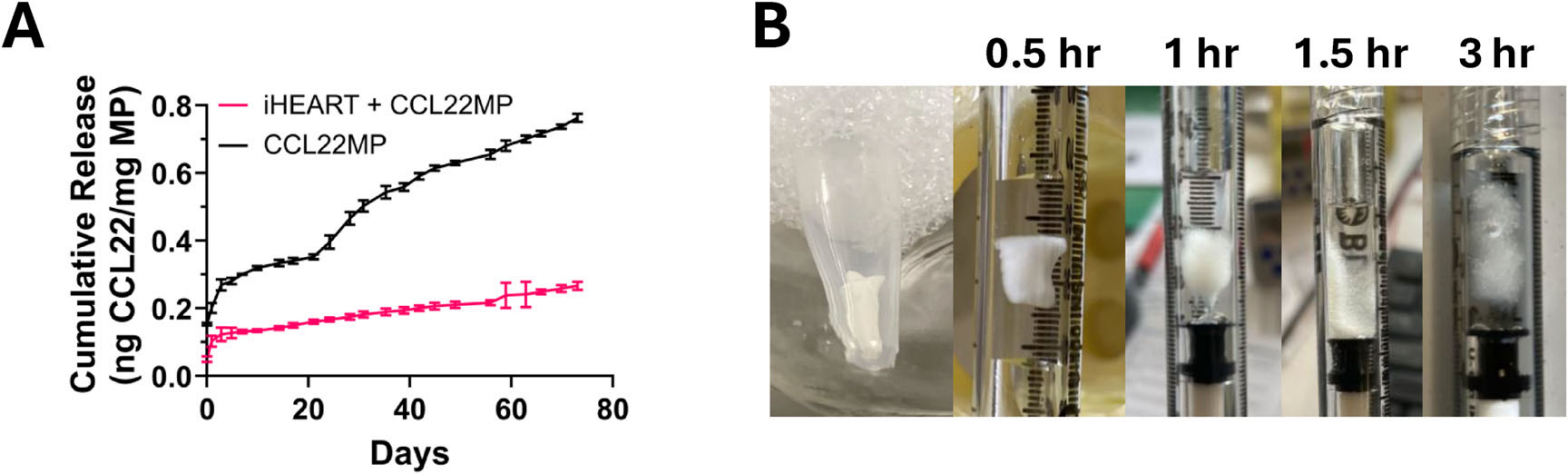
Characterization of injectable HEART. (A) Cumulative release of injectable HEART (iHEART) in comparison to free CCL22MP. (B) Images from a rehydration study of iHEART show full rehydration after 3 h.

We next evaluated injection variability by encapsulating AlexaFluor680‐labeled dextran within the microparticles (AF680MP) and formulating iHEART to contain 20 mg/mL (nominal dose) and 100 mg/mL (high dose) of MP (Figure 3). Injections into agar (Figure 3A) reveal high variability of AF680MP delivery for the high dose formulation and comparable payload delivery between formulations (Figure 3B). Further analysis of the syringe body and needle following injection reveals a significant loss (∼70%–80%) of AF680MP in the needle and syringe body for the high‐dose formulation. Because of the improved reproducibility of injection and comparable delivery of payload, 20 mg/mL of MP was selected for subsequent ex vivo biocompatibility experiment with a Langendorff apparatus (Supplementary Figure S1). The heart's contractility, relaxation, and workload were not significantly affected by iHEART injection.

**FIGURE 3:**
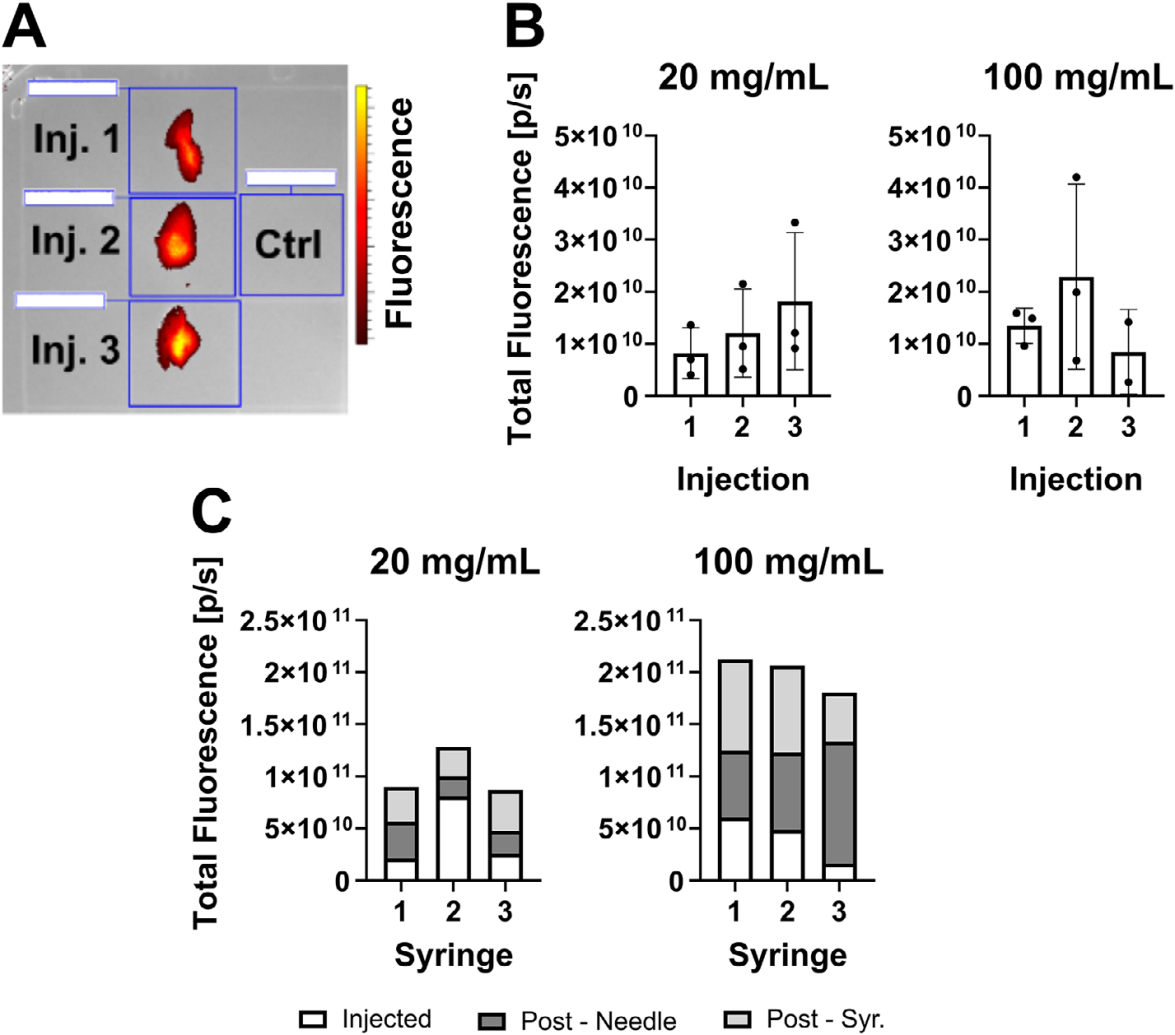
MP delivery variance following iHEART injection. MP concentration for optimal suspension in iHEART was determined empirically. (A) Representative fluorescent images of hydrogel and AlexaFluor680‐labeled Dextran MP repeatedly injected (Inj. #) into agar or BlankMP control (Ctrl). (B) Quantification of total fluorescence in agar injection sites, normalized to gel and BlankMP auto‐fluorescence. (C) Comparison of total MP injected into agar (White) and the fraction remaining in the needle (Dark Grey) or syringe body (Light Grey). Data shown in B represent mean ± SD of three (3) repeated injections, in series, from the same syringe.

### 3.3 Biocompatible, iHEART Exhibits High Retention of MP in the Myocardium but Does not Yield Functional Improvement in an Ischemia Reperfusion MI Model

We next evaluated the retention of MP using iHEART in the myocardium of healthy rats. Fluorescent AF680MP were suspended in hydrogel (Gel+MP) or saline (Saline+MP) and compared to BlankMP suspended in iHEART (Vehicle Control). It was found that 14 days after injection, the Gel+MP group had significantly improved retention of MP as compared to the Saline+MP group (Figure 4A). Qualitatively, suspension of MP within iHEART produced a high fluorescent intensity peak followed by an ellipsoidal radiance of fluorescence while MP suspended in saline produced a diffuse fluorescent peak (Figure 4B). Following these positive results, we conducted a study in a clinically relevant, ischemia‐RI model of MI (Figure 5). Both BlankMP and CCL22MP iHEART showed comparable cardiac function to healthy controls, but there was no detectable difference in cardiac function between the two groups. Further, there were positive trends between the untreated group (MI control) and iHEART treatment groups, but the differences were not statistically significant.

**FIGURE 4:**
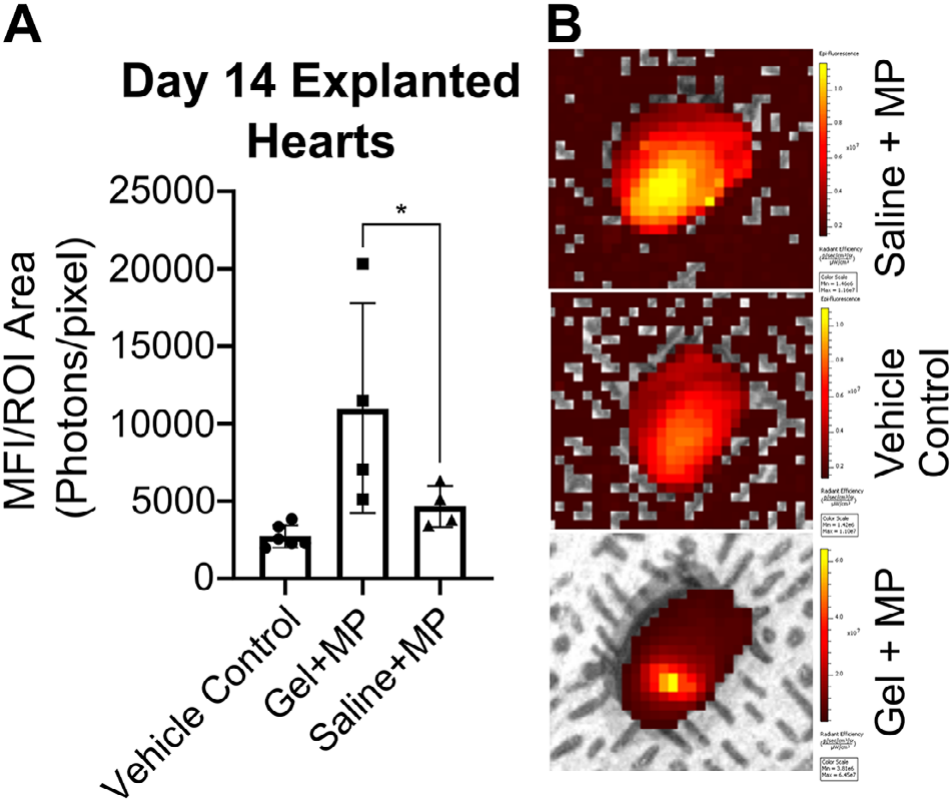
iHEART improves cardiac retention of MP compared to free injection. (A) Mean fluorescent intensity (MFI) normalized to the region of interest (ROI). (B) Representative fluorescent images—note: the scale bar is different for each image. Scale ranges are as follows: Saline+MP (1.46e6 – 1.16e7, 2e6); Gel+MP (3.81e6 – 6.45e7, 2e7); Vehicle Control (1.42e6 – 1.10e7, 0.2e6) (min—max, major increment). Data represents mean ± SD for n = 4–6/group, *p < 0.05.

**FIGURE 5:**
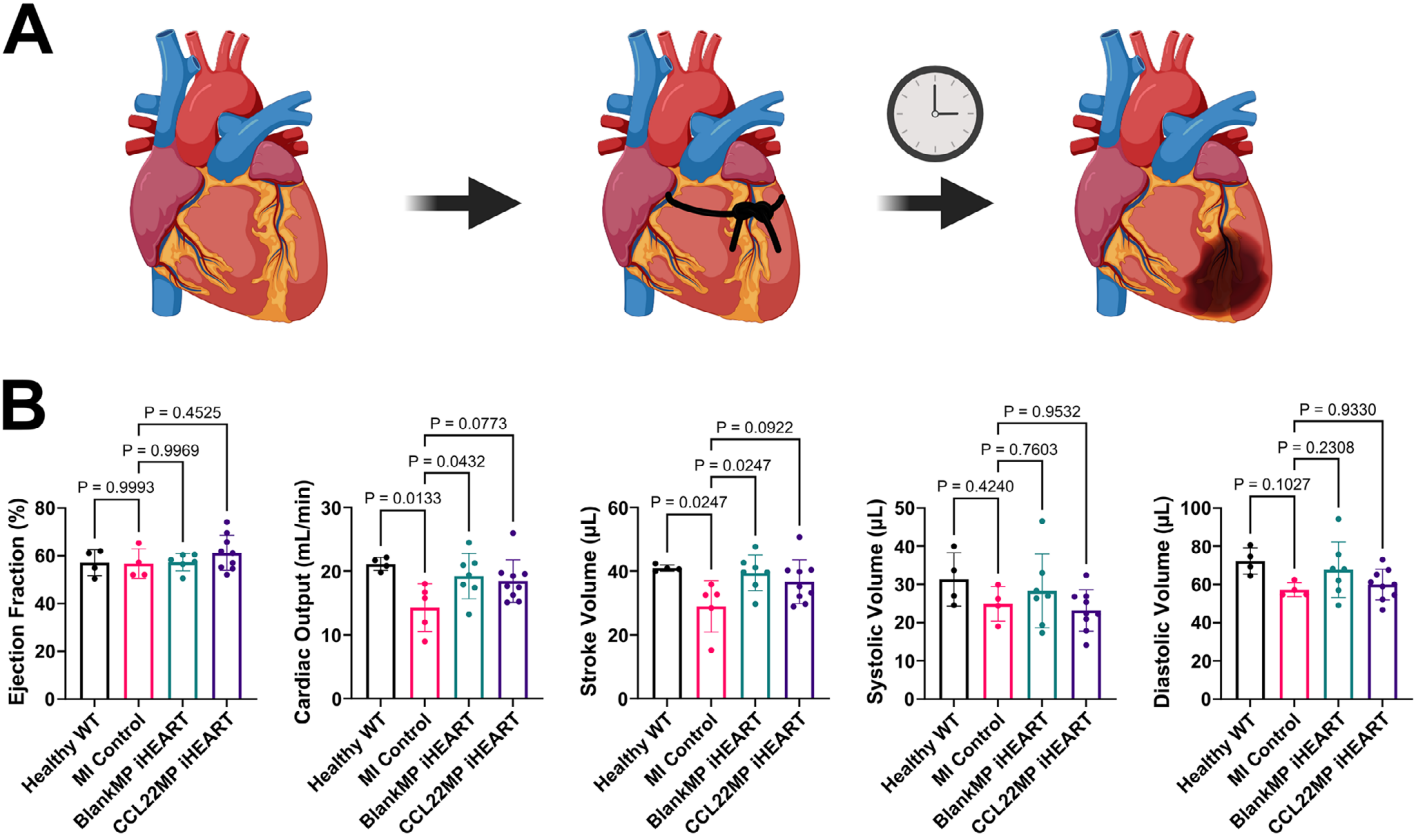
CCL22MP iHEART therapy did not significantly improve cardiac function following ischemia‐reperfusion MI. (A) Ischemia‐reperfusion MI model was performed by ligating the LAD artery for 30 min after which time the ligating suture was removed and the tissue allowed to reperfuse. Created with BioRender.com. (B) Echocardiographic cardiac functional metrics recorded 7 days following ischemia‐reperfusion MI show BlankMP and CCL22MP have comparable performance to healthy wildtype control, but do not produce statistically significant improvements relative to MI injury control. Data represents mean ± SD for n = 4 to 9.

### 3.4 Formulation of a Topical and Tissue Adhesive Hydrogel for Localized CCL22MP Delivery to the Heart

While an injectable hydrogel is highly translatable to the clinic, the delivered volume of gel is roughly five‐fold smaller in comparison to the topical formulation. Therefore, in some cases topical delivery may be superior. To that end, we trialed several tissue‐adherent hydrogels of various chemistries for localizing the delivery of CCL22MP to the heart. Specifically, we investigated a hydrogel formed from HEMA‐functionalized hyaluronic acid, which cross‐links and forms covalent adhesions to the tissue in response to light (UV Gel), as well as three FDA‐approved hydrogel sealants that are approved for use in cardiovascular applications—Coseal, Artiss, and Tisseel. Each hydrogel cross‐links within 5 min, retains MP following cross‐linking, and forms a uniform suspension of MP (Figure 6A). Tisseel, Artiss, and the UV gel each retained their form and retained MP for at least 13 days (Figure S2). Variable degradation was observed for the adhesive hydrogel candidates, in which the UV gel lasted >32 days, Tisseel began to show signs of degradation at ∼25 days, Artiss degradation occurred after ∼15 days, and Coseal degradation occurred after ∼6 days (Figure 6B). Notably, Coseal exhibited significant swelling prior to its dissolution (Figure S2). Accordingly, Coseal exhibited comparable release of CCL22 to freely suspended MP while the other hydrogels exhibited slight suppression of CCL22 release (Figure 6C). Prior to selecting the leading candidate gel for in vivo experimentation, we also evaluated the ex vivo cardiac tissue adhesion and retention of MP was evaluated for the adhesive hydrogels (Figure 7). Artiss and Tisseel displayed increased fluorescence, indicating improved MP retention. However, only Tisseel produced reduced variance resulting in statistically significant differences in fluorescence. Based on these data, we determined that Tisseel would be the best candidate for application in vivo.

**FIGURE 6:**
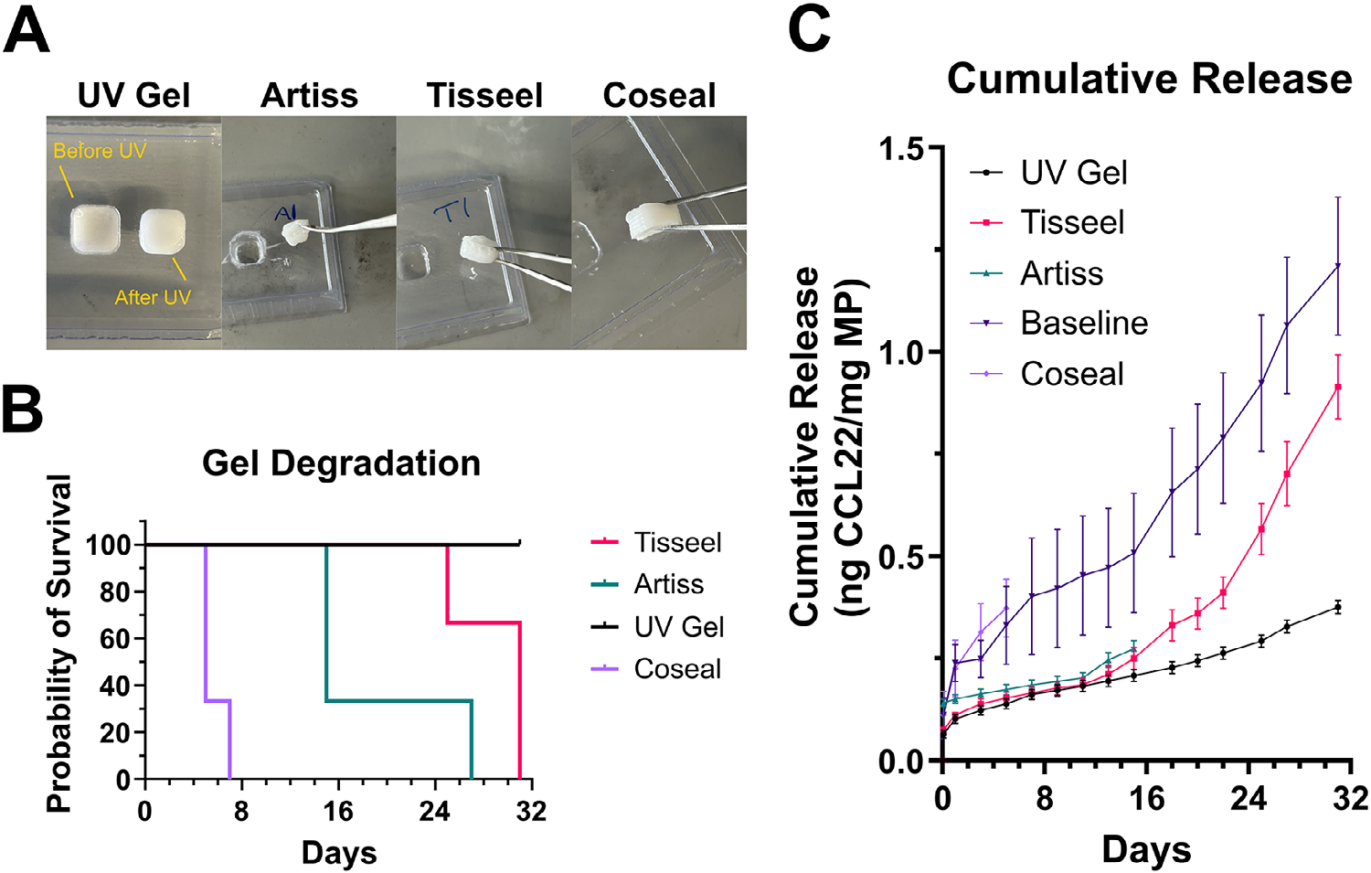
Formulation of Adhesive Hydrogels for CCL22MP Localization to the Heart. (A) Representative images of adhesive hydrogels loaded with MP after initiation of cross‐linking, all hydrogels are colorless, and MP (10 mg/mL) are white. (B) Evaluation of hydrogel degradation rates, a gel was considered fully degraded when it was indistinguishable from the saline it was suspended in. (C) Release profiles of CCL22MP suspended in adhesive hydrogels. Release is concluded following hydrogel dissolution. Data shown represent mean ± SD for N = 3 individual experiments.

**FIGURE 7:**
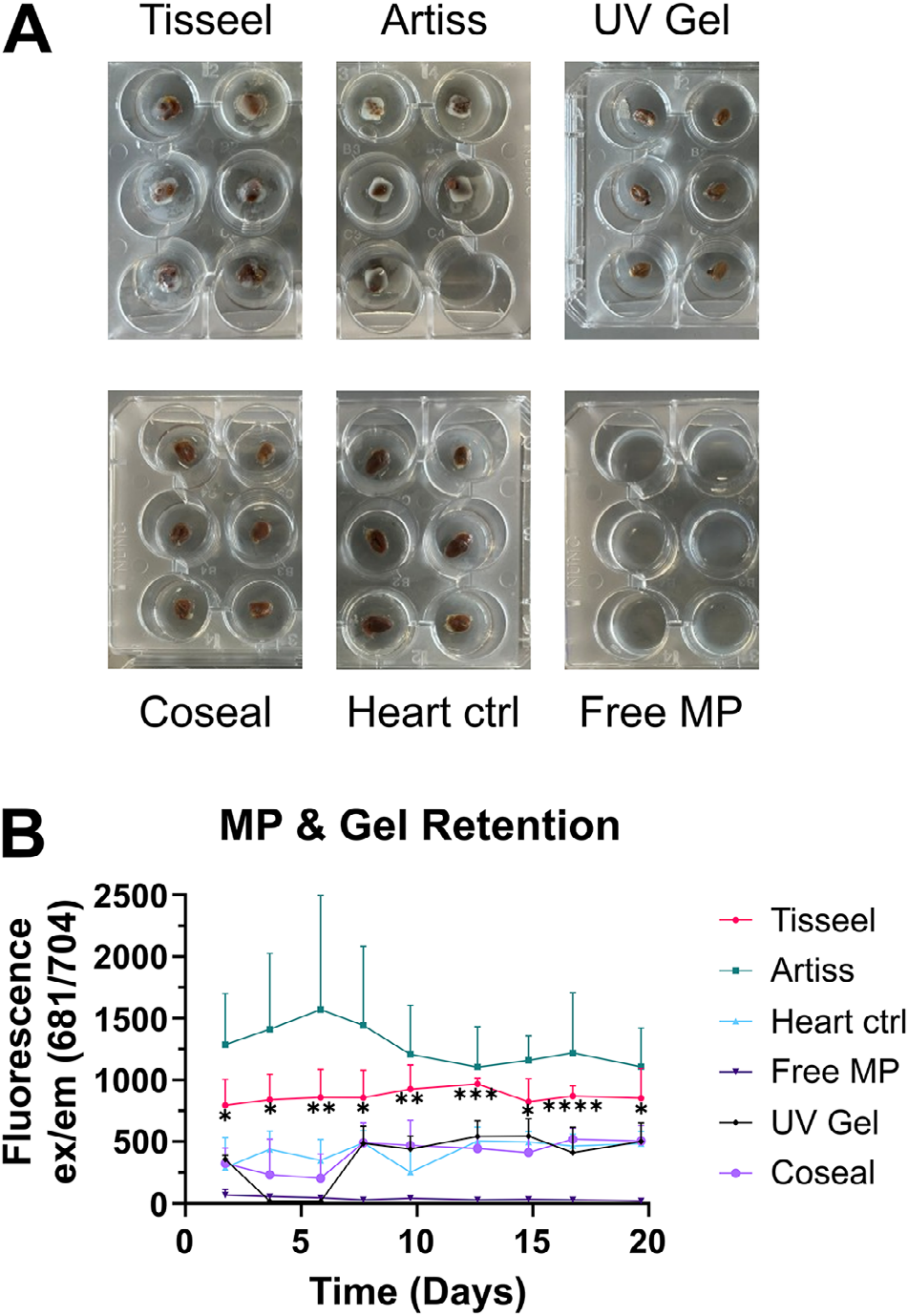
Ex vivo tissue retention of adhesive hydrogels. (A) Images of adhesive hydrogels applied to ex vivo hearts for 20 days and controls (heart alone, freely suspended AF680 MP. (B) Quantification of AF680 MP fluorescence over time. Data shown represent mean + SD for N = 5 to 6 independent experiments. *p < 0.05, **p < 0.01, ***p < 0.001, ****p < 0.0001.

### 3.5 CCL22MP Suspended Within Tissue Adherent Hydrogels Reduce Transplant Ischemia Injury in Syngeneic Transplants

Based on our prior studies testing CCL22MP localization to the heart to treat cardiac injury, we endeavored to determine how CCL22MP treatment may affect a decentralized injury to the entire heart, as seen in transplant ischemia. The study design is described in Figure 8A,B shows a representative image of Tisseel + MP applied to a donor heart following its transplantation. Histologic sectioning and trichrome staining 30 days after transplant ischemia (Figure 8C,D) show CCL22MP localization in Tisseel hydrogel significantly reduces fibrosis area in the donor hearts relative to injury and BlankMP controls. FoxP3 staining of the donor hearts from POD30 (Figure 8E,F) show CCL22MP in Tisseel hydrogel significantly increased FoxP3^+^ Treg in the donor hearts relative to injury and Blank MP controls.

**FIGURE 8:**
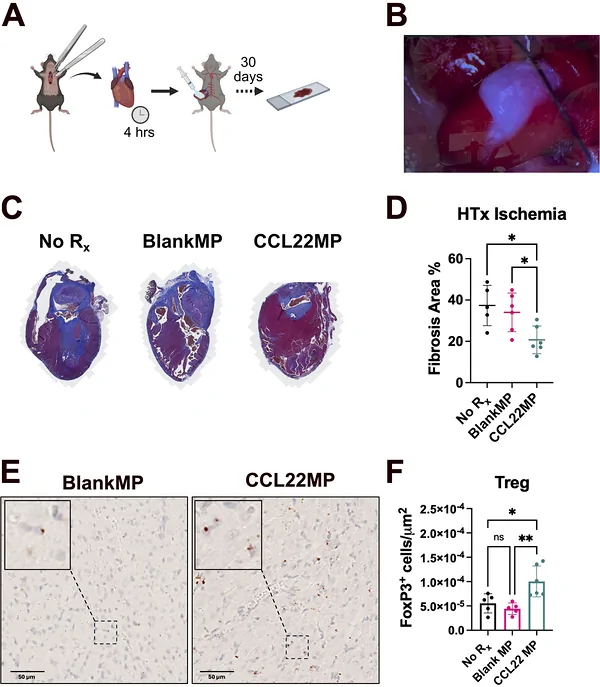
CCL22MP suspended in tissue adhesive tisseel hydrogel reduces transplant ischemic injury and increases local treg. (A) Study scheme. Syngeneic donor hearts were subjected to 4 h ischemia before transplantation into recipient mice. After 30 days the transplanted hearts were harvested for histologic sectioning. Created with BioRender.com. (B) Representative surgical image of MP‐loaded Tisseel hydrogel applied, topically, to a transplanted heart. (C) Representative histologic sections of hearts receiving no treatment (No R_x_), Tisseel + BlankMP control (BlankMP), or Tisseel + CCL22MP treatment (CCL22MP). (D) Quantification of fibrotic tissue area %. (E) Representative IHC detection of FoxP3^+^ cells (brown nuclear DAB staining) across the transplant sections at POD30. Insets show high‐magnification of FoxP3^+^ cells. Scale bars: 50 µm. (F) Quantification of FoxP3^+^ cells across the transplant sections at POD30 normalized to the total area. Data shown represents mean ± SD for n = 5 to 6/group. *p < 0.05, **p ≤ 0.01.

Flow cytometric characterization of infiltrating immune cells (see Figure S3 for gating strategy) at an early timepoint (POD5), however, did not reveal any significant change in the frequency or cellular concentration of immune cells (Treg, macrophages, Pan T cells, or B cells; Figure S4). Assessment for expression of proteins indicative of pro‐inflammatory (CD86, iNOS) versus reparative (ST2, CD301b, Arg1) functions did not reveal any early differences driven by CCL22MP in treatment of ischemic heart tissues (Figure S4). Together, these data demonstrate that topical CCL22MP delivery in Tisseel significantly enriches intragraft FoxP3^+^ Treg and reduces fibrotic area by POD30, without detectable changes in immune cell frequency or activation state at POD5. This temporal pattern is consistent with the sustained release kinetics of CCL22MP, in which a local chemokine gradient accumulates over weeks rather than acting acutely in the first days following administration.

## 4 Discussion

In these studies, we evaluated multiple **H**ydrogels to **E**nhance or **A**ttract **R**egulatory **T** cells (**HEART**) and quantified potential benefits in several models of cardiac ischemic injury. Prior research has documented that Treg are necessary to stabilize the infarcted heart [23, 54]. Studies investigating Treg‐secreted soluble factors (i.e. IL‐10 [28, 55], Areg [56]) or systemic mobilization of Treg from lymphoid tissues [31] report reductions to cardiac damage following MI. To our knowledge, however, therapeutics that aim to attract Treg directly to the heart have not been developed. Moreover, there are no reported studies that aim to understand the limitations of Treg therapy by examining cardiac injury models of varying severity. To translate our Treg‐attracting [34] CCL22 releasing MP for cardiac delivery, we developed and characterized multiple formulations of HEART that leverage different approaches for cardiac localization. Subsequent application of various HEART formulations demonstrates CCL22MP treatment provides significant reduction to cardiac injury for injury models that induce significant cardiac damage, specifically MI induced by permanent ligature of the LAD artery and cardiac transplant ischemia. Importantly, we do not observe a statistically significant benefit in a transient, ischemia‐reperfusion‐injury model of MI. Together, these findings suggest Treg chemoattraction through CCL22MP treatment has the greatest potential benefit after a significant cardiac event.

Considerable research has been conducted to understand the importance of a well‐coordinated immune response following MI. Like other tissue injuries, the body's innate immune system—neutrophils, monocytes, macrophages, etc.—infiltrates the injured tissue and acts to clear inflammatory cell debris [16, 21, 22]. While the clearance of cell debris is necessary for adequate repair, these cells can also become activated by local inflammatory markers and perpetuate damage to the myocardium [57, 58]. In addition to innate responders, the adaptive immune system is also activated. Specifically, CD4^+^ T cells have been reported to appear shortly after MI, and they have been implicated as a contributor to infarct spreading [59]. Because Treg are the natural regulator of both innate and adaptive immunity in a highly sophisticated and dynamic way [60], they have gained considerable interest. Sophisticated mechanistic studies that leverage genetic ablation of Treg or inhibit their immunosuppressive function reveal Treg have several beneficial roles following acute MI. Treg have been shown to induce infiltrating macrophages toward an M2 repair phenotype and amplify the secretion of collagen resulting in improved scar formation and reduced risk of myocardial rupture [54]. In addition, Treg reduce inflammatory cell infiltrate, cardiac apoptosis, and the concentrations of harmful matrix metalloproteinase enzymes that induce pathologic tissue remodeling [26]. Thus, amplifying the local population of Treg has significant potential to improve repair after MI.

Chemoattraction of Treg to inflamed tissues through CCL22MP administration is well‐characterized from our prior studies. Treg chemoattraction in response to CCL22 was originally observed in cancer resulting in CCL22‐releasing tumors evading clearance by the immune system [61, 62, 63, 64]. Our first reported formulation of CCL22MP demonstrated that Treg will traffic to the site of injected CCL22MP [34]. In the years since developing CCL22MP, we have demonstrated CCL22MP reduces disease severity and amplifies tissue repair in several models of inflammatory disease including dry‐eye disease [38], transplantation rejection [37], and periodontitis [39]. In our prior studies with CCL22MP, the daily quantity of CCL22 released by the MP ranges from tens of picograms to nanograms, but they all demonstrate continuous, linear release that is necessary to establish a constant gradient of CCL22. The various formulations we developed of HEART exhibit suppressed release of CCL22 relative to free CCL22MP release. The hydrogels used to formulate HEART were fabricated from many synthetic and biopolymers including poly(NIPAAm), hyaluronic acid, PEG‐NHS, and Fibrin/Thrombin. Functionalized hyaluronic acid was used to create the injectable formulation of HEART, as well as the tissue adhesive UV‐Gel. Suspension of CCL22MP into these hydrogels produced a 50% reduction in the release rate of CCL22 in early time points and a greater deficit amounting to 80% reduced release at longer timescales. Hyaluronic acid exhibits a negative charge under physiologic conditions [65], so it is possible positively charged CCL22 may interact with the hyaluronic acid, potentially slowing the release rate. Further, CCL22 release from MP suspended within Artiss and Tisseel hydrogels is comparably slowed. The positively charged thrombin enzyme converts negatively charged fibrinogen into a relatively more positively charged fibrin hydrogel, which should not interact with CCL22 and may even accelerate its release rate [66]. Similarly, the NHS groups on the PEG polymer in the Coseal hydrogel react quickly to form covalent bonds and are not likely to interact with CCL22 during its release. Notably, the release rate of CCL22 from Coseal most closely matches freely suspended CCL22MP release. Taken together, the most likely explanation for the observed reduction in CCL22 release rate from these HEART formulations is that the hydrogel adds an additional physical, diffusional barrier from the cross‐linked hydrogel. Thus, suppressed release does not occur from MP suspended in Coseal hydrogel because of its high degree of swelling which is inversely correlated to cross‐link density [67].

In our prior studies with CCL22MP, the particles were administered to tissues that facilitate high retention (subdermal, intramuscular) or were co‐localized to an organ as a packed pellet. Adopting the former approach is not viable because freely suspended MP are poorly retained in the myocardium [68]. In our prior experience, the latter approach requires large quantities of MP to be administered and has potential to cause localized tissue irritation from an overabundance of acidic degradation products [69], and it also lacks the ability to be localized for a sustained duration. Thus, we innovated by creating HEART and leveraged multiple modalities to enable tissue localization and retention. In our pilot study with a permanent ligature MI model, we used a thermally responsive hydrogel. We have previously administered this thermogel, loaded with MP, to create a conformable and highly retained delivery system in the paranasal sinuses [70, 71]. While our pilot study showed CCL22MP treatment was efficacious, we did not directly quantify the retention of the thermogel as this material is nondegradable and therefore not a candidate for clinical translation. The positive results prompted the development of a more clinically relevant biodegradable and injectable formulation of HEART (iHEART) in a pre‐clinical MI model. Evaluation of in vivo retention demonstrated that iHEART facilitated high retention of suspended MP, but we did not observe efficacy in the ischemia‐reperfusion‐injury model of MI. From Figure 4B, the diffusion of the fluorescent tracer from the MP occurs along the latitudinal axis, which is consistent with the muscle fiber orientation [72]. In comparison to topical delivery, less MP are delivered in direct injection, and it is possible that released CCL22 does not effectively access the lymphatics system, which is damaged following MI [73], or secondary lymphoid organs, while topical delivery may access these tissues more easily to mobilize Treg. Because the damage induced by the immune response during RI is directly related to the extent of damage [10], the lack of detectable efficacy may also be a consequence of marginal potential benefit.

Further, our evaluation of a topically applied, tissue‐adhesive formulation of HEART demonstrated a significant reduction in cardiac damage in a highly reproducible transplant model of severe ischemia‐reperfusion‐injury. In cardiac transplantation, graft fibrosis restricts cardiac function and is associated with cardiac allograft vasculopathy (CAV), ultimately leading to poor graft survival [74, 75]. Given that active cardiac functional parameters cannot be directly quantified in this model, we utilized fibrotic tissue area as a primary metric of repair and graft preservation, a strategy consistent with work by Zhang et al. [76], who demonstrated that reducing post‐MI fibrosis via engineered FAP CAR Tregs restores cardiac output. Topical CCL22MP administration significantly reduced graft fibrosis (Figure 8D) and increased the local Treg population (Figure 8F) by POD30, demonstrating a positive correlation between Treg accumulation and reduced fibrosis, and supporting Treg recruitment as a promising therapeutic approach to prolong graft survival. These findings are complemented by our initial myocardial infarction model, in which topical CCL22MP delivery in a thermally responsive hydrogel reduced infarction size and improved ejection fraction, systolic volume, and diastolic volume (Figure 1).

Early flow cytometric characterization of donor hearts at POD5 did not reveal significant differences between CCL22MP‐ and BlankMP‐treated grafts in the frequency or number of infiltrating Treg, macrophage, T cell, or B cell populations, nor in the expression of markers indicative of pro‐inflammatory (CD86, iNOS) or reparative (ST2, CD301b, Arg1) function (Figure S4). This is consistent with the sustained‐release design of the platform, in which the local CCL22 gradient accumulates over weeks rather than acting acutely; therefore, the intervention is not anticipated to exert its principal effect within the first postoperative days. We further note that this cohort (n = 4 per group) was not powered to resolve modest differences. Local immune modulation after injury is nonetheless transient, and denser early sampling may be required to capture it. For example, in a cardiotoxin‐induced skeletal muscle injury model, local delivery of the same CCL22MPs drove transient modulation across days 3, 5, and 7, characterized by increased ST2 expression on Treg and increased frequencies of Ly6C^lo^ macrophages, peaking at day 3^37^.

Taken together, this work suggests that localized Treg recruitment via topical CCL22MP delivery is an effective therapeutic strategy for preserving cardiac tissue following ischemic injury, particularly in models of more severe damage. Across our pilot permanent ligature MI model and our clinically relevant transplant ischemia model, topical administration of HEART significantly reduced cardiac damage. Crucially, in the transplant ischemia model, this reduction in cardiac injury was accompanied by a significant enrichment of intragraft Treg at POD30. While the injectable formulation (iHEART) enhanced MP retention without impairing baseline cardiac function, delivery in the ischemia‐reperfusion MI model did not significantly improve cardiac function, likely reflecting the reduced MP payload and restricted access to lymphatics or secondary lymphoid tissue compared to topical administration. Future studies utilizing genetic depletion models (such as FoxP3‐DTR mice [51]) will be essential to confirm the therapeutic necessity of recruited Treg, while CCR4 knockout models will help elucidate the mechanisms through which CCL22 acts on other CCR4‐expressing populations. Ultimately, these findings suggest topically applied CCL22MP‐loaded hydrogels as a promising therapeutic strategy to address severe cardiac ischemic injury.

## Author Contributions

**Matthew A. Borrelli**: conceptualization, data curation, formal analysis, investigation, methodology, visualization, writing – original draft, writing – review and editing. **Alexis T. Reese**: conceptualization, data curation, formal analysis, investigation, methodology, visualization, writing – original draft, writing – review and editing. **Jordan J. P. Warunek**: conceptualization, data curation, formal analysis, investigation, methodology. Betsy A. Varghese—data curation, investigation. **Canxing Lin**: methodology, data curation, investigation. **Jeffery J. Baust**—methodology, data curation, investigation. **Brenda Mcmahon**: data curation, formal analysis. **Steven R. Little**: funding acquisition, resources, supervision, writing – review and editing. **Hēth R. Turnquist**: funding acquisition, resources, supervision, writing – review and editing.

## Funding

This work was supported by funding from National Institutes of Health (R01HL122489, F31HL170514, and T32HL076124), the Department of Defense (RT200012, RT230018, and PR230360), and the American Heart Association (23PRE1026713). These funders played no role in study design, data collection, analysis and interpretation of data, or the writing of this manuscript.

## Conflicts of Interest

The authors declare no conflicts of interest.

## Supporting information




**Supporting File**: adhm71861‐sup‐0001‐SuppMat.pdf.

## Data Availability

The data that support the findings of this study are available on request from the corresponding author. The data are not publicly available due to privacy or ethical restrictions.
