# Supplementary material for: Topically Delivered Treg‐Attracting Microparticle‐Hydrogel Composites Recruit Regulatory T Cells and Limit Cardiac Injury After Severe Ischemia
Source: Adv Healthc Mater. 2026 Oct 18;15(42):e71861. doi: 10.1002/adhm.71861 (PMC13768202; doi:10.1002/adhm.71861)
Supplement: Supplementary file 1 [file ADHM-15-0-s001.pdf]

Supporting Information

Topically delivered Treg-attracting microparticle-hydrogel composites recruit regulatory T cells and limit cardiac injury after severe ischemia

Matthew A. Borrelli[1,*], Alexis T. Reese[1,*], Jordan J. P. Warunek[2,3,4], Betsy Ann Varghese[2,3], Canxing Lin[2,3], Jeffery J. Baust[5], Brenda McMahon[6], Steven R. Little[1,4,7,8,9,10,11,#], Hēth R. Turnquist[2,3,4,11,#]

*These authors contributed equally to this work.

#Corresponding Author Emails: Heth R. Turnquist (het5@pitt.edu); Steven R. Little (srlittle@pitt.edu).

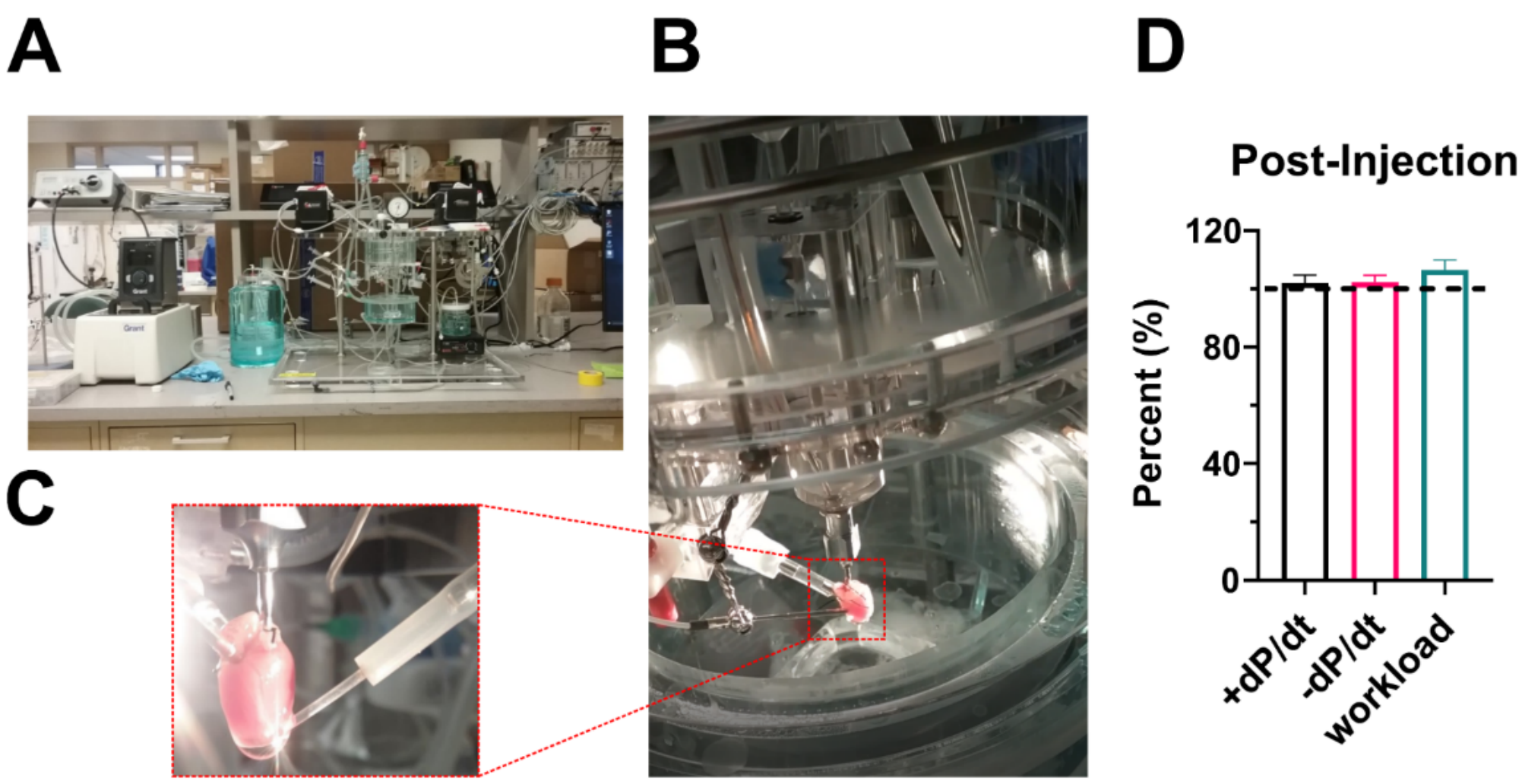

Supplementary Figure 1 – iHEART Injection Does Not Affect Cardiac Function

(**A,B**) Images of Langendorff apparatus, (**C**) Zoomed image showing probe positioning on excised heart. (**D**) Quantification of contractility (+dP/dt), relaxation (-dP/dt), and calculated workload are unchanged relative to baseline readings recorded prior to injection. (n = 4/treatment group and data are mean ± SD.

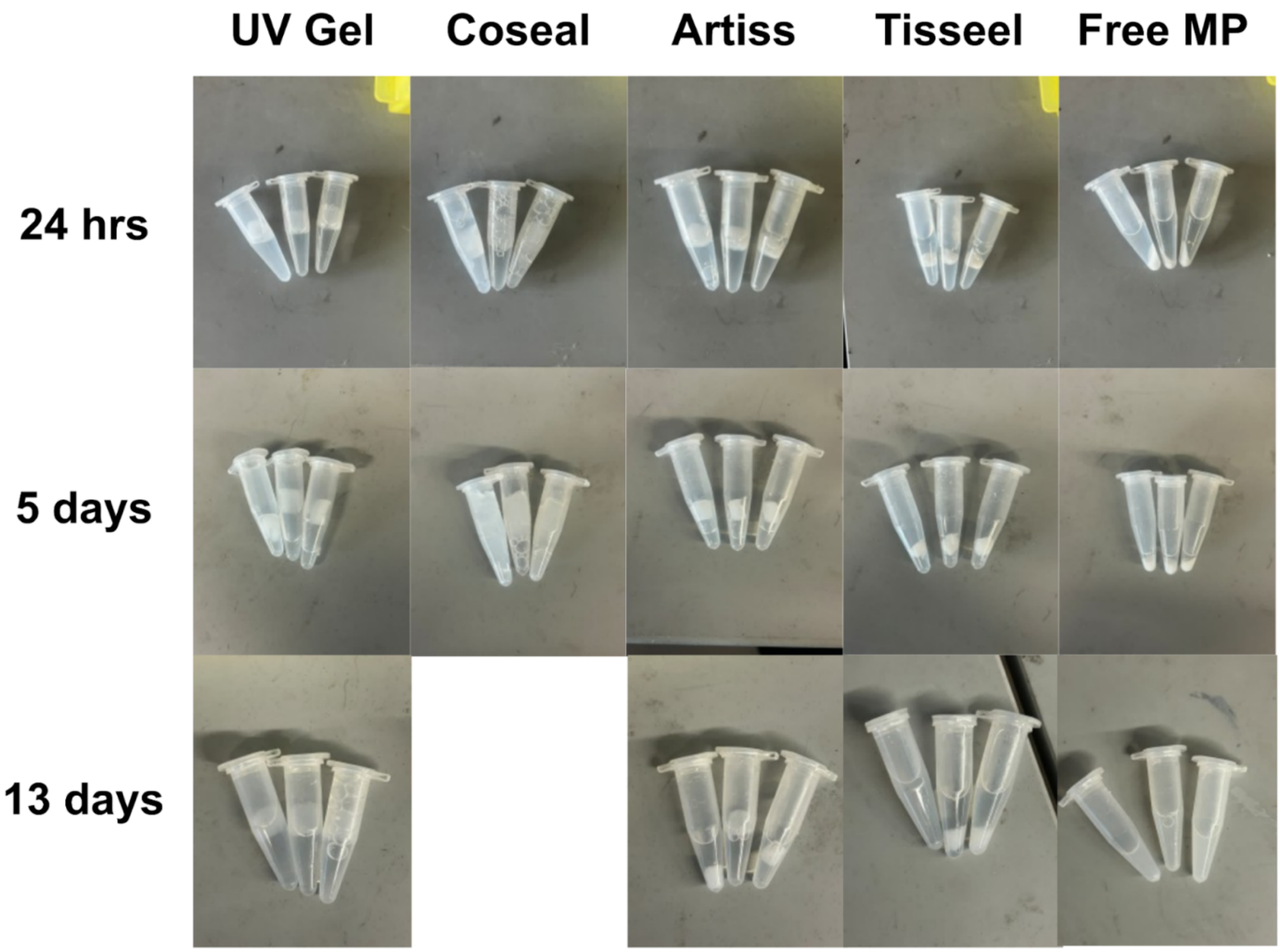

Supplementary Figure 2 – Adhesive Hydrogel Morphology After Incubation in Saline

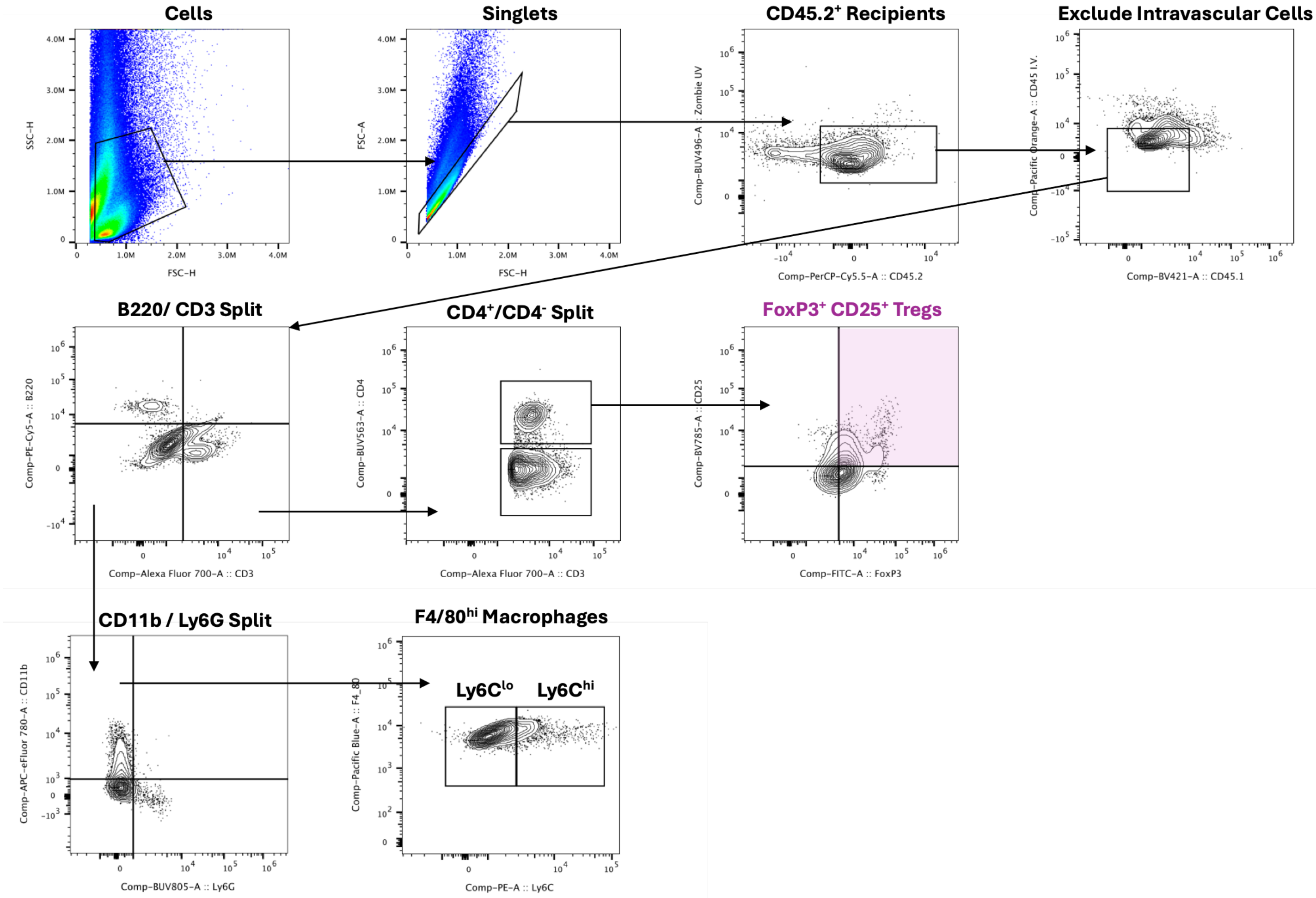

Supplementary Figure 3 – Flow Cytometry Gating Strategy for Characterization of Graft Infiltrating Immune Populations

Representative gating scheme used to identify recipient (CD45.2$^+$) derived populations isolated from digested CD45.1$^+$ donor hearts at POD5. This gating strategy allows quantification of immune cell infiltration (CD45.2$^+$) of CD45.1$^+$ ischemic heart transplants showing quantification of CD45.2$^+$ Pan CD3$^+$ T cells, Treg (FoxP3$^+$ CD25$^+$ CD4$^+$ CD3$^+$) and B220$^+$ B cells, macrophages (F4/80^{hi} CD11b$^+$ Ly6G$^-$ B220$^-$ CD3$^-$ CD45.2$^+$). Macrophages were also assessed for expression of proteins indicative of pro-inflammatory (CD86, iNOS) versus reparative (ST2, CD301b, Arg1) functions.

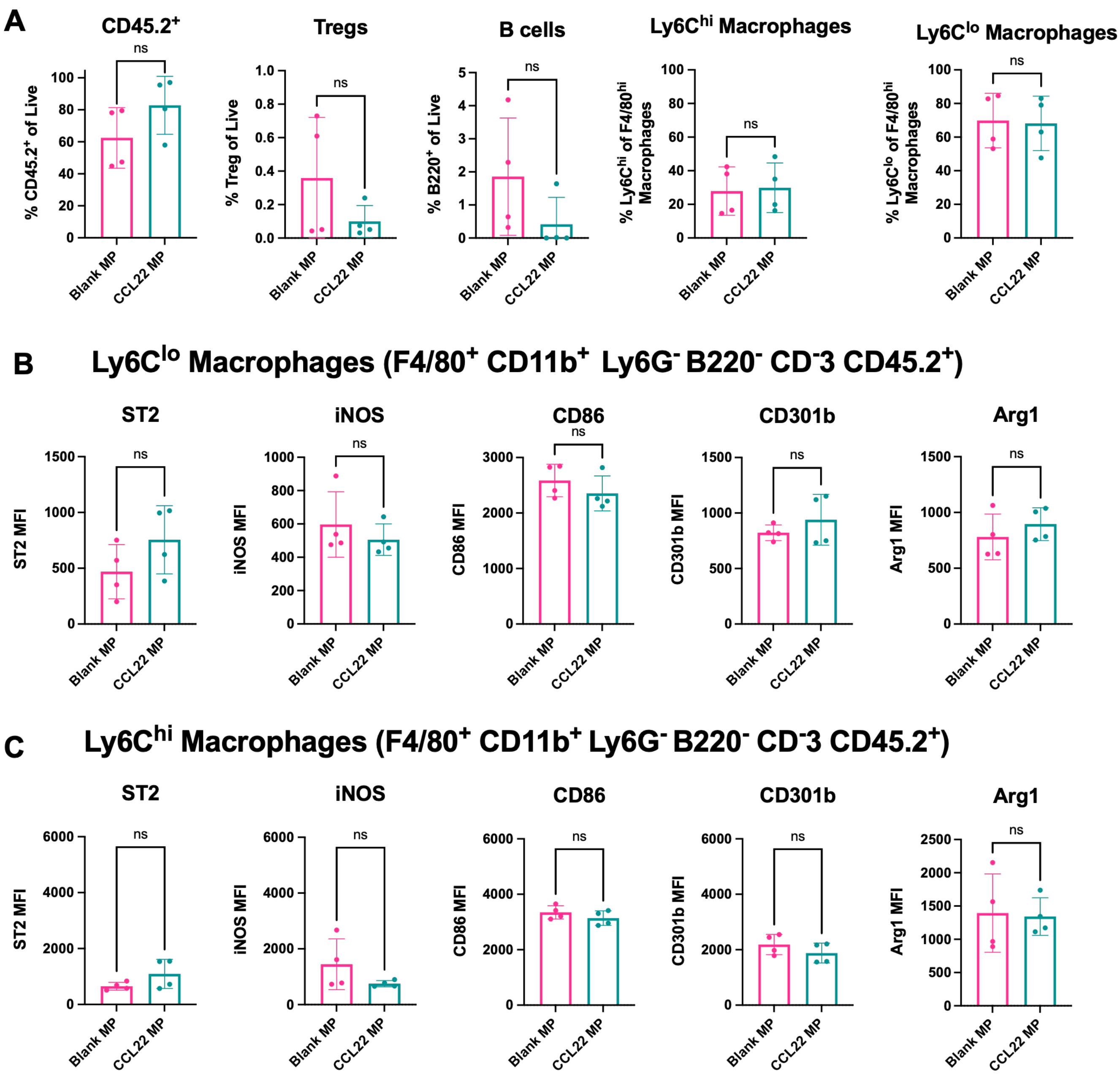

Supplementary Figure 4 – Characterization of Infiltrating Immune Populations at POD5 and Treatment with BlankMP or CCL22MP In Tisseel Hydrogel.

(A) Detected frequency of indicated infiltrating immune populations isolated at POD5 from digested $CD45.1^+$ ischemic syngeneic grafts. Intravascular immune cells were excluded with i.v. CD45. (**B**-**C**) Functional phenotype based on expression level (MFI) of phenotypic and/or effector molecules by $Ly6C^{lo}$ and $Ly6C^{hi}$ macrophages. Data represent mean ± SD (n = 4 / group).
